# Vomocytosis of *Cryptococcus neoformans* cells from murine, bone marrow-derived dendritic cells

**DOI:** 10.1371/journal.pone.0280692

**Published:** 2023-03-16

**Authors:** Noah Pacifici, Melissa Cruz-Acuña, Agustina Diener, Allen Tu, Neeraj Senthil, Hyunsoo Han, Jamal S. Lewis

**Affiliations:** 1 Department of Biomedical Engineering, University of California—Davis, Davis, CA, United States of America; 2 J. Crayton Pruitt Family Department of Biomedical Engineering, Gainesville, FL, United States of America; University of Kent, UNITED KINGDOM

## Abstract

*Cryptococcus neoformans* (CN) cells survive within the acidic phagolysosome of macrophages (MΦ) for extended times, then escape without impacting the viability of the host cell via a phenomenon that has been coined ‘vomocytosis’. Through this mechanism, CN disseminate throughout the body, sometimes resulting in a potentially fatal condition—Cryptococcal Meningitis (CM). Justifiably, vomocytosis studies have focused primarily on MΦ, as alveolar MΦ within the lung act as first responders that ultimately expel this fungal pathogen. Herein, we hypothesize that dendritic cells (DCs), an innate immune cell with attributes that include phagocytosis and antigen presentation, can also act as ‘vomocytes’. Presciently, this report shows that vomocytosis of CN indeed occurs from murine, bone marrow-derived DCs. Primarily through time-lapse microscopy imaging, we show that rates of vomocytosis events from DCs are comparable to those seen from MΦ and further, are independent of the presence of the CN capsule and infection ratios. Moreover, the phagosome-altering drug bafilomycin A inhibits this phenomenon from DCs. Although DC immunophenotype does not affect the total number of vomocytic events, we observed differences in the numbers of CN per phagosome and expulsion times. Interestingly, these observations were similar in murine, bone marrow-derived MΦ. This work not only demonstrates the vomocytic ability of DCs, but also investigates the complexity of vomocytosis regulation in this cell type and MΦ under multiple modulatory conditions. Understanding the vomocytic behavior of different phagocytes and their phenotypic subtypes is needed to help elucidate the full picture of the dynamic interplay between CN and the immune system. Critically, deeper insight into vomocytosis could reveal novel approaches to treat CM, as well as other immune-related conditions.

## Introduction

Numerous pathogens have demonstrated means for persistence, dissemination, and infection within their mammalian hosts. The fungal species *Cryptococcus neoformans* (CN) is an opportunistic pathogen that causes an infectious disease called ‘Cryptococcosis’ that predominantly affects immunocompromised patients—primarily those afflicted with HIV/AIDS [1–3]. This infection may progress to a condition called Cryptococcal Meningitis (CM), where CN establishes an infection in the central nervous system (CNS). Globally, there are estimated to be 223,100 CM cases and 181,000 deaths annually [1].

*Cryptococcus neoformans* cells typically enter the host through the lung via inhalation. Subsequently, the fungal pathogen disseminates from the lung into other tissues, including the CNS [2, 4]. The three proposed mechanisms that facilitate CN entry into the CNS are paracytosis, transcytosis, and hitchhiking. In the latter, CN are suggested to cross the blood brain barrier (BBB) by ‘hitchhiking’ within host phagocytes [5, 6]. This is known as the ‘Trojan Horse’ hypothesis. *Kechichian et al*. showed that depletion of alveolar macrophages (MΦ) in mice significantly reduces cryptococcal dissemination to the CNS [7]. *Charlier et al*. later showed that infecting naïve hosts with CN-infected monocytes significantly increases CN accumulation in the brain, compared to infecting with CNs directly [8]. Recently, *Gilbert et al*. also linked CN dissemination (muscular and CNS) to length of time residing within phagocytes in an *in vivo* zebrafish model, supporting the Trojan Horse mechanism of CN transport [9].

Further, Cryptococcal cells have been shown to escape from MΦs by inducing expulsion whilst leaving the phagocyte unharmed through a phenomenon called ‘vomocytosis’ (non-lytic exocytosis) [3, 10–12].

Like most cell types, MΦs regularly perform exocytosis to recycle membrane components and excrete various factors [13–15]. However, vomocytosis is a unique form of exocytosis whereby these immune cells (over 5 to 20 hours) expel large pathogenic particulates that they are otherwise programmed to be retained and digested. Studies have identified intracellular, physicochemical, and immunological cues in CN-infected MΦs that are linked to this mechanism. As MΦs perform vomocytosis, actin rapidly and transiently polymerizes in a cage-like structure around the phagosome, which then fuses with the plasma membrane [16]. These cages may be a post-phagosome permeabilization attempt by the MΦ to inhibit CNs’ escape, as inhibiting actin polymerization has been shown to increase vomocytosis occurrence. In addition, CN disrupts phagolysosomal maturation, as characterized by the rapid removal of the early phagosome markers Rab5 and Rab11 [17]. Some reports have suggested that there is alkalinization of, and abnormal calcium ion levels in phagosomal compartments that contain the live pathogen [17–21]. Furthermore, the addition of weak bases to CN-infected MΦs has been shown to modulate vomocytosis occurrence from MΦs [18, 21]. Moreover, *Gilbert et al*. showed that pharmacological inhibition of ERK5 increases vomocytosis occurrence [9].

Further, the immune state of infected MΦs has been suggested to also influence this phenomenon. A recent study by *Seoane et al*. discovered that viral exposure to either measles or human immunodeficiency virus (HIV) were both shown to significantly boost expulsion rates of CN cells from MΦs [22]. Moreover, other factors known to elicit an antiviral response—the TLR3 agonist poly(I·C) and type I interferons, IFN-α and IFN-β—all similarly increase MΦ vomocytosis events. Another study investigated how different T cell effector-induced phenotypes can impact the expulsion rates of infected J774 MΦs [23]. Prior to infection, these cells were treated with cytokines inducing T effector cell-induced phenotypes Th1 (IFN-γ and TNF-α), Th2 (IL-4 and IL-13), or Th17 (IL-17). The Th1 and Th17 subtypes of J774 cells showed diminished intracellular CN proliferation and increased vomocytosis rates, whilst the Th2 group displayed increased intracellular CN proliferation and reduced vomocytosis occurrence.

Taken altogether, studies have given some clarity on the influence of intracellular, physicochemical, and immune states on vomocytosis. However, they focus entirely on MΦs, which are only a single cell type in an army of immune cells. Moreover, *Yang et al*. recently discovered the occurrence of this phenomenon in neutrophils [24].

Like MΦs and neutrophils, dendritic cells (DCs) have the unique ability to phagocytose particulates, including pathogens. More importantly, DCs link the innate and adaptive arms of the immune system. These innate immune cells are key for maintaining a balance between the host defense against pathogens and protection of “self” antigens of host cells and tissues [25–27]. Dendritic cells detect invading pathogens due to their constituent sensors (e.g. Toll-like receptors [TLRs]) [28, 29]. They communicate the presence of pathogens to the adaptive immune system, thereby initiating long lasting, antigen-specific responses. Migration of DCs to T cell-rich regions is critical here and is mainly regulated by the chemokine receptor CCR7 [30–32] and CCL21 [33–35]. Following DC migration to secondary lymphoid organs, lymphocytes are subsequently activated and induced to proliferate and become potent effector cells (e.g. helper T cells) [25]. Interestingly, other innate immune cell types can traffick via lymphatics [32, 36] and perform antigen presentation [37–39]. While these cell types have some promising capability in lymphatic migration and antigen presentation, their abilities are limited in comparison to DCs, which are recognized as the primary antigen presenting cell type whose dominant function is to traffick to the lymph nodes (LNs) to present foreign material to LN-resident T cells.

During cryptococcal infections, murine, bone marrow-derived DCs are known interact with CN cells and have been demonstrated to phagocytose CN cells following opsonization with complement or antibody [40]. *Hole et al*. demonstrated the ability of murine, bone marrow-derived DC lysosomal extract to cause morphological changes in CN and kill the pathogen *in vitro* via oxidative and non-oxidative mechanisms [41]. *Artavanis-Tsakonas et al*. showed that CN-containing murine, bone marrow-derived DC phagosomes have an impaired CD63 recruitment, indicative of a distinct phagosomal compartment composition that may affect the outcome of antigen processing and presentation [42]. However, to the best of our knowledge, the ability of DCs to expel CN from their phagosome via vomocytosis has not been investigated thus far.

We hypothesized that DCs could perform vomocytosis, especially given that they share much of the same vacuolar machinery as their phagocytic relatives, MΦs and neutrophils. In this present study, the DCs used are defined as GM-CSF differentiated cells from murine, bone marrow-derived progenitors and characterized by presence of CD11c and absence of F4/80 surface markers. Herein, we endeavored to document vomocytosis from DCs—a key player in bridging the innate and adaptive immune response. Further, we investigated the effect of CN infection ratio, presence of CN capsule, drug manipulation of the phagosome conditions and actin polymerization on vomocytosis from DCs. The overall effect of the immune state on vomocytosis from both DCs and MΦs was also assessed. Finally, we characterized vomocytosis based on multiple outcomes—rate of vomocytosis occurrence, timing of expulsion, and number of internalized CN prior to expulsion. We believe that further investigation of CN’s complex interactions with different phagocytic cell types will act as a step towards elucidating the complex story of cryptococcal infections and underlying mechanisms of vomocytosis.

## Materials and methods

### Bone marrow-derived DC and MΦ culture

All animals were maintained and used in accordance with NIH guidelines and approved by UC Davis Institutional Animal Care and Use Committee (approval number 21840). Mice were housed four per cage, 20–26°C ambient temperature, 12-hour light/dark cycle, with ad libitum access to food and water. Animals were monitored by husbandry staff at least once every day, with monthly healthcare checks by a veterinarian. Consistent with the recommendations of the Panel on Euthanasia of the AVMA, mice in this study were euthanized by lethal dose of CO_2_ asphyxiation followed by cervical dislocation. This method of euthanasia is not painful and minimizes any discomfort experienced by the animal. Primary DCs and MΦs were obtained from the bone marrow of C57BL/6 mice as described in previous studies [43–45]. Growth media consisted of DMEM/F-12 1:1 with L-glutamine (Cellgro, Herndon, VA), 10% fetal bovine serum, 1% sodium pyruvate (Lonza, Walkersville, MD), 1% nonessential amino acids (Lonza, Walkersville, MD), 1% penicillin/streptomycin (Cytiva, Marlborough, MA) and 20 ng/mL GM-CSF (R&D Systems, Minneapolis, MN) (DC media) or 10% L929 conditioned media (MΦ media) and incubated at 37°C and 5% CO_2_. For conciseness, media containing all necessary growth factors and added reagents for the necessary cell type will be denoted as ‘complete media’. Unless otherwise noted we used DC and MΦ on day 10 of their respective cultures.

### Cell phenotype validation via flow cytometry

On day 6 of DC or MΦ culture, cells were characterized by measuring the presence of phenotype-specific surface markers, with antibodies against F4/80 [APC, BM8 Clone] (eBioscience, San Diego, CA) and CD11c [PE-Cy7, HL3 Clone] (BD Pharmingen, San Diego, CA) via an Attune Nxt Flow Cytometer (Life Technologies, Carlsbad, CA).

### Cell phenotype validation via RNA analysis

On day 10 or 11 of DC or MΦ culture, the contents of cells were extracted via application of TRIzol (Thermo Fisher Scientific, Waltham, MA). To isolate RNA from samples, a chloroform solvent extraction was performed according to manufacturer instructions. Next, RNA was purified using a kit, RNA Clean & Concentrator with DNAse (Zymo Research, Irvine, CA). The DNA Technologies Core at UC Davis assessed RNA quality (score>7.0), performed Batch 3’Tag-Seq library preparation, and sequenced using an Illumina NextSeq sequencer (Illumina, San Diego, CA). For analysis, reads were trimmed, aligned, and quantified for gene counts using OmicSoft software (Qiagen, Hilden, Germany). Dimensional reduction for principal component analysis (PCA) plots was also performed in the OmicSoft software.

### Dendritic cell and MΦ polarization validation

Dendritic cells and MΦs were seeded on 12 well plates (0.5 million cells/ well [DCs] and 0.25 million cells/ well [MΦs]) in complete media containing polarizing agents. The difference in seeding density was due to spreading ability, with MΦs being much more elongated than DCs and therefore taking up more surface area per cell. For inflammatory activation, cells were treated with LPS (100 ng/ml) from *Escherichia coli* O111:B4 (Sigma, St. Louis, MO). For anti-inflammatory polarization DCs were treated with 1uM of dexamethasone (DEX; Alfa Aesar, Tweksbury, MA) and MΦs with IL-4 (20ng/ml) and IL-13 (20ng/ml; denoted ’IL4/13’ for brevity; R&D Systems) in MΦ media. After a 48-hour incubation period, the expressions of cell surface markers were determined via flow cytometry using antibodies against F4/80, CD38 [PerCP-eFluor 710, 90 Clone] (eBioscience), Arginase 1 [PE, A1exF5 Clone] (eBioscience), and iNOS [PE-Cy7, CXNFT Clone] (eBioscience) for MΦs and antibodies against CD11c, MHCII [Alexa-Fluor 488, M5/114.15.2 Clone] (BD Pharmingen), CD80 [APC, 16-10A1 Clone] (BioLegend, San Diego, CA), and CD86 [PE, GL1 Clone] (BD Pharmingen) for DCs. Additionally, an LPS activation resistance test was performed by adding LPS (100 ng/ ml) to DCs and MΦs previously treated with tolerogenic polarization agents for an additional 48 hours. Subsequently, the expressions of the same cell surface markers were quantified using flow cytometry.

### Effect of infection rate on vomocytosis

Dendritic cells and MΦs were seeded on 24 well plates (75,000 cells/ well [DCs] and 50,000 cells/ well [MΦs]) in complete media and incubated at 37°C and 5% CO_2_. Infections with CNs and subsequent time-lapse imaging studies were performed between day 11–15. Wildtype CN H99 and the acapsular mutant *cap59* CN (both generously gifted by Dr. Angie Gelli, UC Davis, CA) were grown first on yeast extract peptone dextrose (YPD) agar (Thermo Fisher Scientific) followed by transferring a single colony to YPD broth (Thermo Fisher Scientific) shaking at 30°C overnight. The next day, CNs were washed with PBS (x3) via centrifugation. The heat-killed (HK) CN negative control group was prepared by incubating CNs at 70°C on a heat block for 1 hour. Both live and HK CNs were opsonized with 10 ug/ml of the anti-capsular IgG1 monoclonal antibody 18B7 (supplied from both Sigma and the Casadevall Lab, Johns Hopkins University, MD) and 50% human AB serum (Sigma). Opsonized pathogen was co-incubated with phagocytes at a 1:1 or 5:1 CN:phagocyte ratio (c.p.r.) for 2 hours in media with 10% human AB serum. Next, infected culture wells were washed with complete media (x5) to ensure all extracellular CNs were removed. Lastly, complete media was added, and time-lapse imaging was performed on infected cells for 14 hours.

### Effect of drugs on vomocytosis

For drug-treated experimental groups, DCs were infected with CN at a 5:1 c.p.r. using identical methods outlined previously. After the 2-hour phagocytosis and washing step, the wells were treated with DC media containing either 10 uM of chloroquine [CQ] (Thermo Fisher Scientific), 100 nM of cytochalasin B from *Drechslera dematioidea* [CYT low] (Sigma), 4 uM of cytochalasin B (CYT hi), or 100 nM of bafilomycin A1 [BFA] (Sigma). These parameters were selected based on the range of drug concentrations used in prior vomocytosis studies[16, 18, 24, 46]. This step was followed by time-lapse imaging for 14 hours whilst in drug-containing media.

### Effect of polarizing agents on vomocytosis

For experiments studying the effect of phagocyte polarization on vomocytic frequency, DEX or LPS were added to DCs at 48 hours prior to CN infection. For MΦs, IL4/13 or LPS was added to the cells 48 hours prior CN infection. Polarizing agent-containing media was replaced with fresh complete media prior to infection with CN. The infection then proceeded using identical methods as previously outlined.

### Time-lapse imaging

Infected cells were kept at 37°C and 5% CO_2_ in the imaging chamber of the BZ-X Fluorescence Microscope (Keyence, Itasca, IL)_._ Images were taken every 4 minutes for a period of 14 hours and compiled into a single movie file using BZ-X software. Movies were blinded by a third party before manual tracking of CNs and scoring for vomocytosis events by an independent technician. After completing a non-lytic expulsion of at least one CN, the phagocyte was then labeled as a ‘vomocyte’ regardless of the number of expelled fungal cells or subsequent vomocytosis events. After analysis, the percentage of observed CN-containing phagocytes that had performed a vomocytosis event, or ‘% vomocytes’ was recorded as the vomocytosis rate. For the presentation and discussion of data in this study, ‘% vomocytes’ and ‘vomocytosis percentage’ are used interchangeably.

### Confocal time-lapse and high-resolution imaging

Before infection, DCs were stained with 1,1’-Dioctadecyl-3,3,3’,3’-Tetramethylindodicarbocyanine, 4-Chlorobenzenesulfonate Salt (DiD; 1μM; Thermo Fisher Scientific) and CN were stained with Calcofluor White (CFW; 1mg/ml; Sigma). After 2 hours of infection at a 1:1 c.p.r., the cells were washed 5 times with DC media. Next, 4 hours after washing, the sample was fixed with 2% paraformaldehyde and vomocytosing cells were imaged using the Olympus FV3000 confocal (Olympus Corporation, Westborough, MA) at 60x magnification.

### DC viability

Dendritic cells were seeded on 24-well plates (75,000 cells/ well) in DC media and incubated at 37°C and 5% CO_2_. Cells were co-incubated with CNs opsonized using previously mentioned methods. Extracellular CNs were washed with DC media and 14 hours later cell viability was measured using the CyQUANT lactate dehydrogenase (LDH) cytotoxicity assay (Thermo Fisher Scientific) according to manufacturer’s instructions. Additional details on the viability assays are provided in the Supplementary Information.

### Data and statistical analysis

In each experimental group replicate, 300 randomly selected CN-containing phagocytes from multiple viewing regions were observed and vomocytosis manually quantified. All statistical analyses were performed using GraphPad Prism 9. Data of vomocytosis frequency, timing, and # CN in the different conditions were assessed using a Kruskal-Wallis test corrected for multiple comparisons by false discovery rate (FDR) using a two-stage linear step-up procedure of Benjamini, Krieger and Yekutieli, or unpaired Mann-Whitney test when there were only two groups to compare. Prior to statistical comparison, raw categorical data of vomocytosis occurrence was converted to continuous data by calculation of individual percentage values for each biological replicate. Flow cytometry data was statistically assessed using a one-way ANOVA corrected for multiple comparisons by false discovery rate (FDR) using a two-stage linear step-up procedure of Benjamini, Krieger and Yekutieli or an unpaired t-test when there were only two groups to compare. All data shown include at least three independent experiments. Original time-lapse movies, upon which manual scoring was performed, are freely available upon request. All column graphs, generated on GraphPad Prism 9, display the individual data points, mean, and standard error mean (SEM). All violin plots, generated on R, visualize the individual event data points, mean (red dot), and box plot containing median and interquartile range. All p-values for significant comparisons are listed in **S1 Table**.

## Results

### DC and MΦ phenotype validation

**Fig 1:**
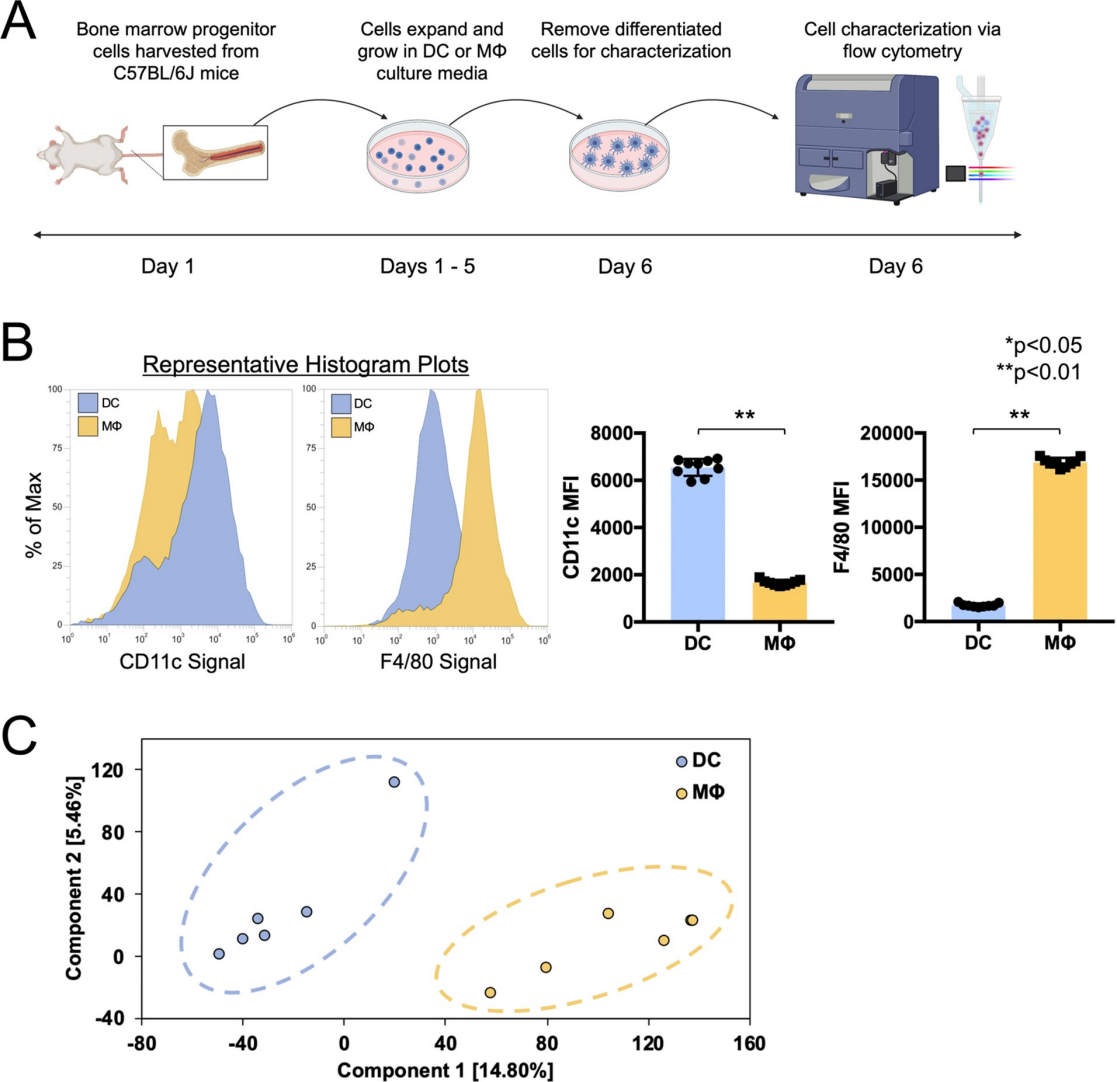
Verification of DC and MΦ phenotypes via F4/80 (MΦ marker) and CD11c (DC marker) flow cytometric analysis and RNA expression analysis. (**A**) Schematic of flow cytometry experiment. Bone marrow progenitor cells were obtained from C57BL/6J mice and grown in either DC differentiation media (GM-CSF supplemented) or MΦ differentiation media (M-CSF supplemented via L929). On day 6, these cells were stained and analyzed via flow cytometry. (**B**) Flow cytometry data for F4/80 and CD11c identification markers. Representative histogram plots of marker signal are displayed. Column graphs of full flow cytometry data show the mean fluorescence intensity (MFI) for F4/80 and CD11c for DC-differentiated and MΦ-differentiated cultures (N = 3, n = 9, statistical analysis performed using an unpaired t-test). (**C**) PCA plot analysis displaying clustering and separation of DC and MΦ cultures by RNA expression data. (N = 6, n = 6, dimensional reduction performed on OmicSoft software).

The phenotypes of the bone-marrow derived DCs and MΦs were characterized via flow cytometry to confirm that the cell cultures generated with these protocols were authentic. Cells were derived from robust techniques used in prior literature for generation of murine, bone marrow-derived DCs and MΦs via GM-CSF [45] and M-CSF [44] respectively. Moreover, cells derived using the above methods have been widely used in prior studies requiring MΦs for vomocytosis experiments [9, 18, 21], and DCs for Cryptococcal infection experiments [40, 47–49]. Here, we validated the growth factor-derived cells using CD11c as a DC marker, and the MΦ marker, F4/80, via flow cytometry. (**Fig 1A**) Dendritic cell cultures exhibit low F4/80 and high CD11c levels, while MΦ cultures display high F4/80 and low CD11c signal. (**Fig 1B**) Furthermore, RNA sequencing analysis of each cell type display notable clustering and separation by PCA plotting. (**Fig 1C**) These results confirm that the cultures grown are genuine bone marrow-derived DCs and MΦs in line with prior literature [50, 51].

### Vomocytosis from DCs is independent of CN capsule and infection rates, and is comparable to vomocytosis from MΦs

**Fig 2:**
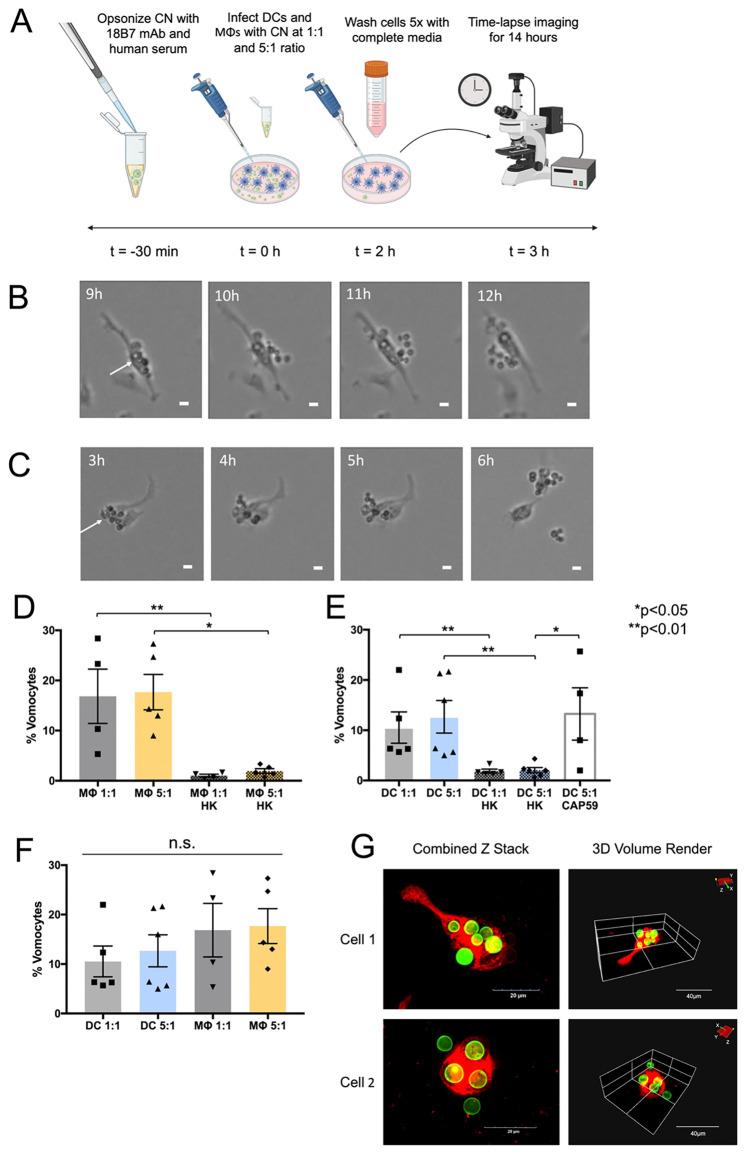
Time-lapse analysis of vomocytosis from DCs. (**A**) Schematic of time-lapse experiment. CN was prepared for phagocytosis by opsonizing with 18B7 mAb and human serum. DCs or MΦs were infected with opsonized CN either at a 1:1 or 5:1 c.p.r. for 2 hours. Following infection, the phagocytes were washed 5x and time-lapse imaged for 14 hours. Representative time-lapse images of DCs performing vomocytosis at (**B**) 1:1, or (**C**) 5:1 c.p.r. (scale bar = 10μm). (**D-F**) Graphs of vomocytosis rates of MΦs and DCs are shown at 1:1 and 5:1 c.p.r., compared to a HK CN control and acapsular *cap59* CN (N≥4 for each condition, statistical analysis performed using a Kruskal-Wallis test corrected for multiple comparisons by FDR using a two-stage linear step-up procedure of Benjamini, Krieger and Yekutieli. Raw categorical data of vomocytosis occurrence was converted to continuous data by calculation of individual percentage values for each biological replicate). **(G)** Confocal images showing instances of DCs (DiD, red) expelling CN (CFW, green). The events are visualized via both a combined Z stack and a 3D volume rendering.

We quantified vomocytic events using time-lapse microscopy and verified their non-lytic nature via cell viability assays. After 2-hour phagocytosis of CN, phagocytic cells were washed for the removal of extracellular CN, and time-lapse imaging experiments were performed during a period of 14 hours (**Fig 2A**). Vomocytosis events, defined as expulsions of CN from host cell while both remain intact, were observed at both CN:phagocyte ratios (c.p.r.) of 1:1 and 5:1, as shown in representative time lapse images (**Fig 2B and 2C**). Confocal time-lapse microscopy videos confirmed vomocytosis events by visually verifying instances of increased CN (green) fluorescent intensity upon uncoupling between DC and CN cells over the course of 8 hours (**S1 Fig**). Overall, vomocytosis occurred at a rate of 17% for MΦs infected at a 1:1 c.p.r., 18% for MΦs infected at a 5:1 c.p.r., 11% for DCs infected at a 1:1 c.p.r., 13% for DCs infected at a 5:1 c.p.r., and 13% for DCs infected with *cap59* CN at a 5:1 c.p.r. (**Fig 2D and 2E**). The vomocytosis rates of acapsular CN-infected MΦs were not shown as this has been investigated previously [10]. For the HK CN control groups, an expulsion rate of only 2% or less was observed for both DC and MΦ groups infected at 1:1 and 5:1 c.p.r. To confirm that these events were indeed non-lytic, DC viability was tested via an LDH assay. We observed no increased toxicity due to CN infection (**S2 Fig**). Notably, the vomocytosis rates observed from DCs were not significantly different from those observed from MΦs at 1:1 and 5:1 c.p.r. (**Fig 2F**). For conclusive visual confirmation of vomocytosis, high resolution confocal microscopy was used to observe two fixed DC cells mid-vomocytosis at 6 hours after infection with CN (**Fig 2G**).

### Disruption of phagolysosomal maturation inhibits vomocytosis

**Fig 3:**
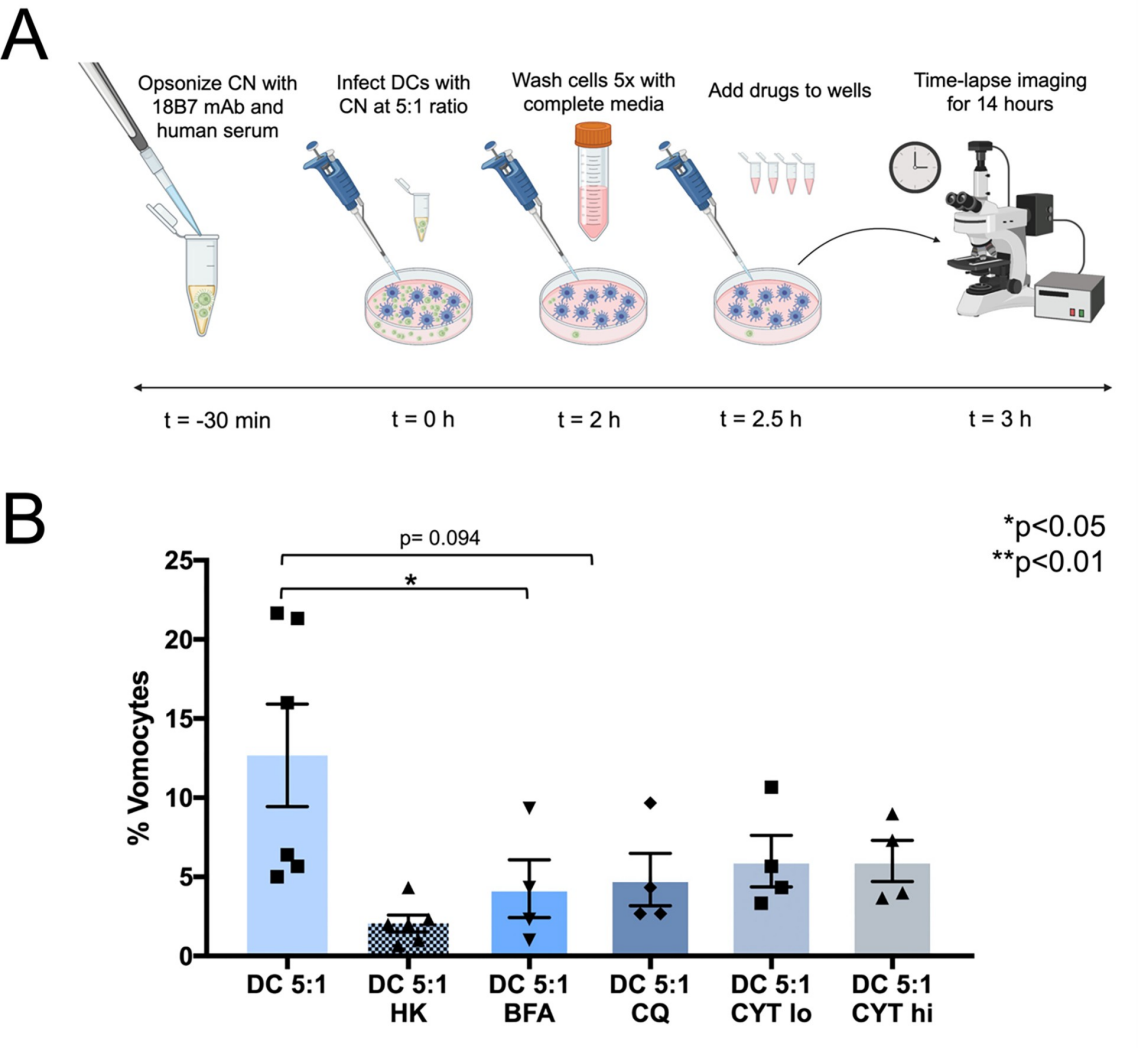
Time-lapse analysis of DC vomocytosis rates under exposure to vomocytosis-modulating drugs. (**A**) Schematic of time-lapse experiment. CN cells were prepared for phagocytosis by opsonizing with 18B7 mAb and human serum. DCs were infected with opsonized CN at a 5:1 c.p.r. for 2 hours. Following infection, the wells were washed 5x, replaced with drug-containing media, and time-lapse imaged for 14 hours. (**B**) Vomocytosis rates of DCs, at a 5:1 infection ratio, are shown in untreated, HK CN, and drug-treated conditions—either BFA, CQ, CYT lo, or CYT hi (N≥4 for each condition, statistical analysis performed using a Kruskal-Wallis test corrected for multiple comparisons by FDR using a two-stage linear step-up procedure of Benjamini, Krieger and Yekutieli. Prior to statistical comparison, raw categorical data of vomocytosis occurrence was converted to continuous data by calculation of individual percentage values for each biological replicate).

Next, DCs were treated with drugs previously documented to affect phagolysosomal maturation, and vomocytosis from MΦs. Namely, CQ, CYT, and BFA were used for these experiments (**Fig 3A**). After assessing potential toxicity to DCs and CNs at the desired concentrations (**S2 Fig**), the individual drugs were added to wells containing CN-infected DCs at a 5:1 c.p.r., just prior time-lapse imaging. All drugs (at the selected concentrations) reduced vomocytosis rates (**Fig 3B**), although CYT and CQ treatments did not show significance in this reduction. For DCs, we observed vomocytosis rates of 4%, 5%, 6%, and 6% for CQ, CYT hi, CYT lo, and BFA respectively. We confirmed that these inhibited rates were not due to the impact of the drugs on immune cell and CN cell viability (**S3 Fig**). Furthermore, in CN-infected DC cultures treated with these drugs, there were no observed increases in toxicity to extracellular or intracellular CNs (**S4 Fig**).

### Treatment of MΦs and DCs with polarization agents alters their immune phenotype

**Fig 4:**
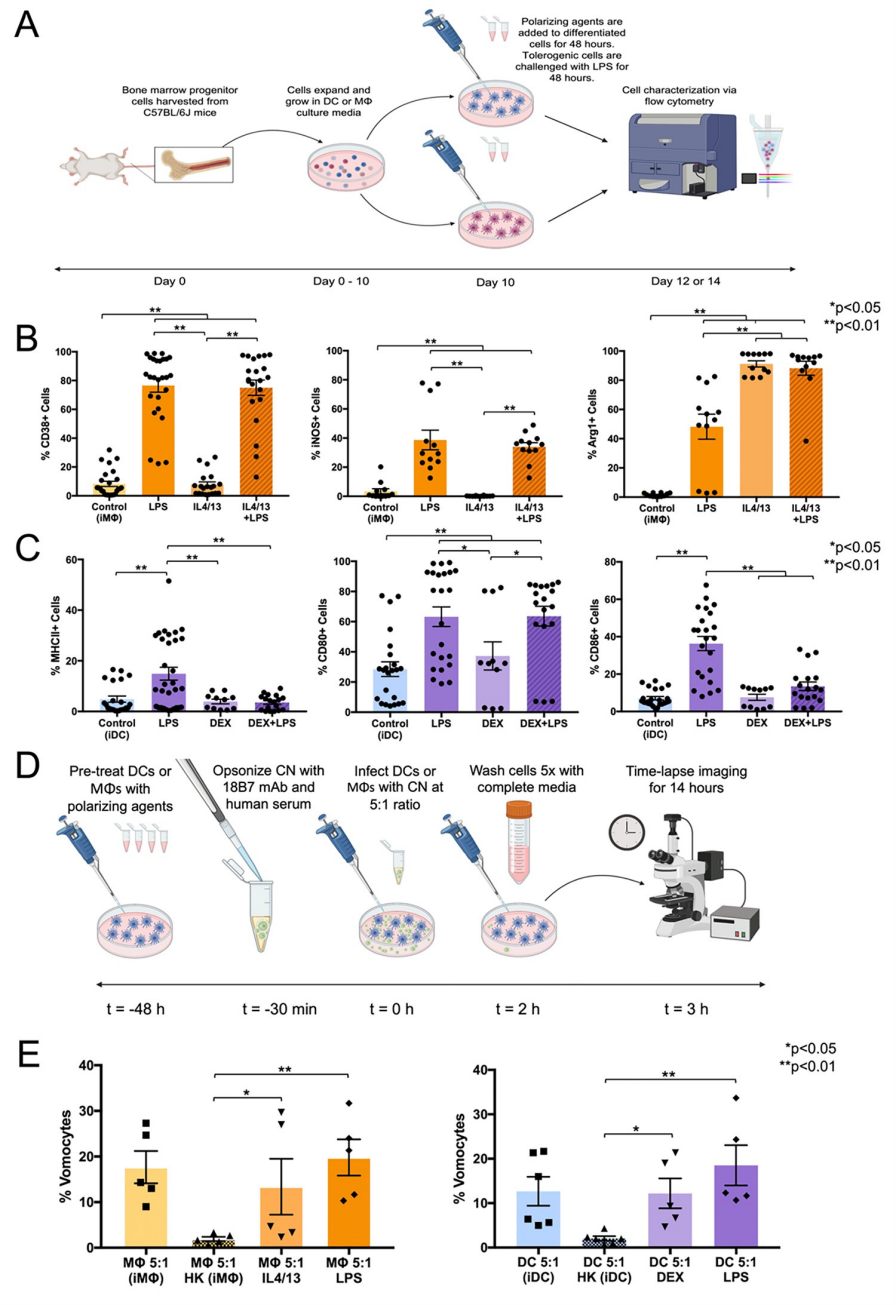
DC and MΦ vomocytosis rates following immune polarization. (**A**) Schematic of flow cytometry experiment. Dendritic cells and MΦs were incubated with polarization agents for 48 hours. Macrophages were polarized using agents LPS or IL4/13 for inflammatory (M1) or tolerogenic (M2) phenotypes, respectively. Dendritic cells were polarized using dexamethasone, or LPS for tolerized or inflammatory phenotypes, respectively. Also, an additional experimental group of tolerized MΦs and DCs were challenged with LPS for another 48 hours. These groups were stained for MΦ immunophenotype markers (CD38, iNOS, and Arg1) or DC immunophenotype markers (MHCII, CD80, and CD86) and analyzed via flow cytometry. (**B**) Confirmation of MΦ immune phenotype characterization by flow cytometry readout of inflammatory activation markers CD38 and iNOS, as well as the M2 marker Arg1. (**C**) Confirmation of DC immunophenotype characterization by flow cytometry readout of maturation markers MHCII, CD80, and CD86. (**D**) Schematic of time-lapse experimental design. Briefly, prior to infection, MΦs and DCs were incubated with polarization agents for 48 hours. Then, CN was prepared for phagocytosis by opsonizing with 18B7 mAb and human serum. Polarized MΦs and DCs were infected with opsonized CN at a 5:1 c.p.r. for 2 hours. Following infection, the phagocytes were washed 5 times and time-lapse imaged for 14 hours. (**E**) Vomocytosis rates of DCs and MΦs at a 5:1 infection ratio are shown for immature (untreated), HK CN, tolerized, and inflammatory states. (N≥4, n≥11 for each condition, statistical analysis performed using one-way ANOVA corrected for multiple comparisons by FDR using a two-stage linear step-up procedure of Benjamini, Krieger and Yekutieli or a Kruskal-Wallis test corrected for multiple comparisons by FDR using a two-stage linear step-up procedure of Benjamini, Krieger and Yekutieli. Prior to statistical comparison, raw categorical data of vomocytosis occurrence was converted to continuous data by calculation of individual percentage values for each biological replicate).

Prior to testing the effect of immune state on vomocytosis, verification of the immunophenotype of MΦs and DCs was performed (**Fig 4A**). In these experiments, the immature MΦ (iMΦ; Control), activated (LPS), anti-inflammatory (IL4/13), and tolerized then challenged (IL4/13 + LPS) conditions were probed (**Fig 4B**). The LPS-treated group displayed significantly higher inflammatory marker expression of CD38 and iNOS compared to the immature, untreated group. Interestingly, the tolerogenic marker Arg1 was also increased on the LPS-treated MΦs; this finding may be due to an inhibitory feedback loop following earlier inflammation in this group. On the other hand, the IL4/13-treated group showed no significant differences for CD38 and iNOS, compared to the immature group, whilst exhibiting higher Arg1 expression than the untreated and LPS groups. Additionally, the tolerogenic group challenged with LPS displayed similarly high levels of Arg1. Additionally, this group had similar expression of CD38 and iNOS markers to the LPS-treated cells.

For DCs, the inflammatory markers MHCII, CD80, and CD86 were analyzed via flow cytometry on CD11c+ gated cells. The immature DC (iDC; Control), activated (LPS), and tolerized (DEX) conditions were analyzed. Additionally, an added DEX-treated DC group was challenged with LPS (DEX + LPS) for an additional 48 hours to test resistance to inflammatory activation. The LPS-treated group displayed significantly higher MHCII, CD80, and CD86 expression compared to the immature untreated group (**Fig 4C**). Meanwhile, the DEX group displayed no significant difference to the immature group on the basis of these inflammatory markers. Furthermore, when tested with LPS after DEX treatment, these tolerogenic DCs displayed significantly lower activation of MHCII and CD86 expressions compared to the LPS-treated group, indicating resistance toward maturation.

### Immune polarization of MΦs and DCs does not affect vomocytosis rate

Next, the vomocytosis rates of polarized MΦs and DCs were tested (**Fig 4D**). For MΦs, the anti-inflammatory (IL4/13) and pro-inflammatory (LPS) conditions showed significantly higher vomocytosis rates than the heat killed control. However, there were no significant differences in rates between the untreated (18%), anti-inflammatory (13%), or pro-inflammatory (20%) MΦs(**Fig 4E, left**). Similarly, DCs polarized to tolerogenic (DEX) and inflammatory (LPS) phenotypes displayed a higher vomocytosis rate than that of the HK CN group. However, the DC vomocytosis rates of the tolerogenic (12%) and inflammatory (19%) groups were not significantly different to each other or the untreated group (13%) (**Fig 4E, right**).

### Infection ratio, drug treatments, and immune polarization affect vomocytosis kinetics

**Fig 5:**
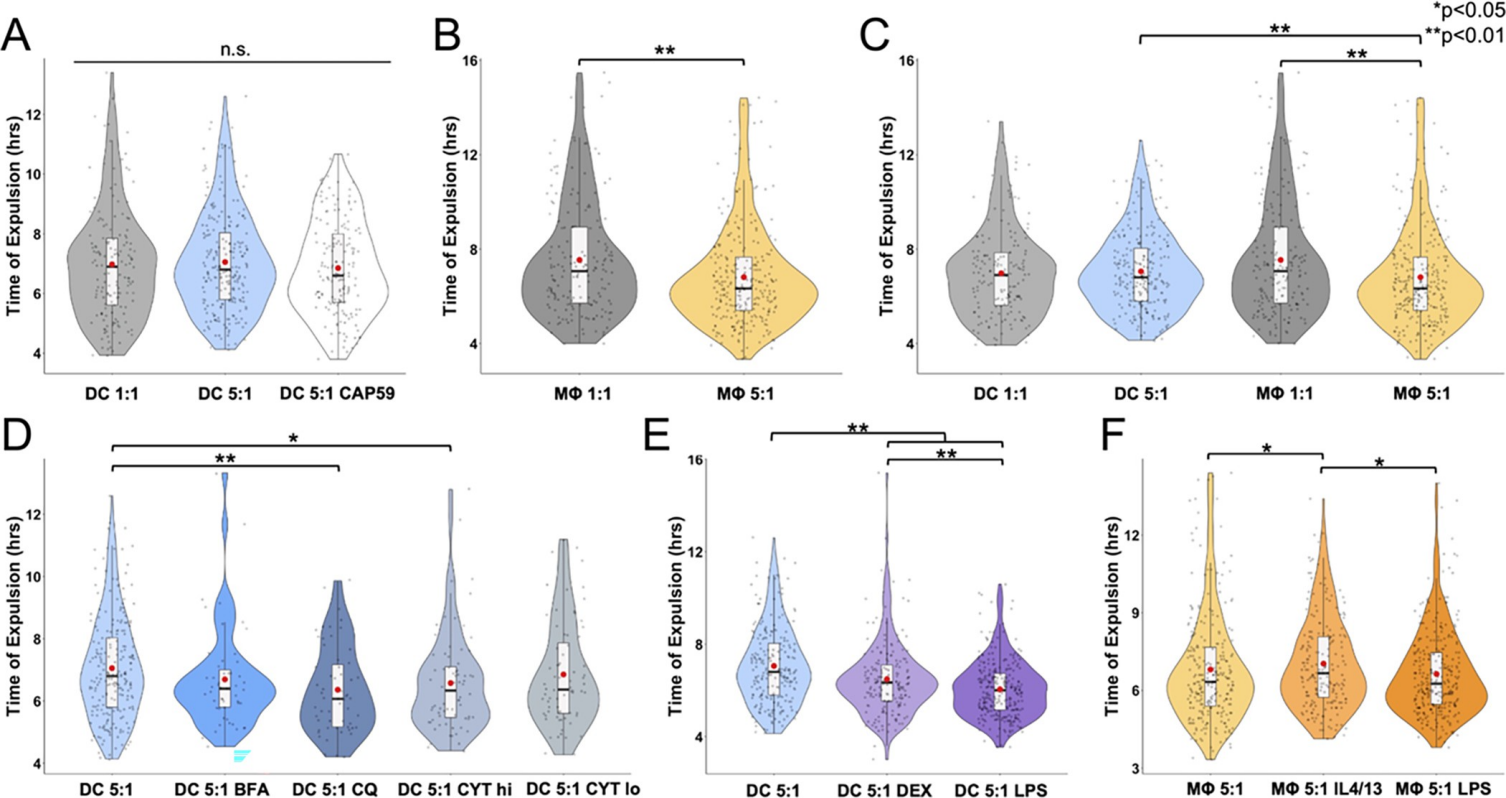
Vomocytosis event timing analysis. Results are displayed in violin box plots with individual dots representing each timing event. The red circle in each plot represents the mean timing occurrence and the black line in the box plot represents the median. (**A**) Violin plot displaying timing of DC vomocytosis events compared between 1:1 and 5:1 c.p.r., as well as 5:1 c.p.r. with *cap59* CN. (**B**) Violin plot of MΦ vomocytosis timing between 1:1 and 5:1 c.p.r. (**C**) violin plot comparing vomocytosis timing between DC and MΦ at different CN infection ratios. (**D**) Violin plot of DC vomocytosis expulsion timing under a 5:1 infection ratio of CN under different drug treated conditions (BFA, CQ, CYT lo, or CYT hi) compared to an untreated control. (**E**) Violin plot of DC vomocytosis timing under a 5:1 CN infection as an immature phenotype (untreated), tolerogenic phenotype (DEX treated), and inflammatory phenotype (LPS treated). (**F**) Graph of MΦ vomocytosis timing under a 5:1 CN infection as an immature M0 phenotype (untreated), tolerogenic M2 phenotype (IL4/13), and inflammatory M1 phenotype (LPS treated) (N≥4, n≥51 for each condition, statistical analyses were performed using an unpaired Mann-Whitney test or a Kruskal-Wallis test corrected for multiple comparisons by FDR using a two-stage linear step-up procedure of Benjamini, Krieger and Yekutieli).

We used time-lapse videos to measure of exact time of expulsion for each vomocytic event. There was no difference in vomocytosis timing for DCs infected with CN at a 1:1 and 5:1 c.p.r., and no difference compared to DCs infected with the *cap59* CN (**Fig 5A**). For MΦs, the 5:1 c.p.r. condition displayed significantly lower average time to expulsion compared to the 1:1 c.p.r. group (**Fig 5B**). Comparing DCs and MΦs, the MΦ 5:1 c.p.r. condition showed a significantly higher time to expulsion than both the DC 5:1 c.p.r. conditions (**Fig 5C**). For the drug-treated DC groups, both the CQ and CYT hi treatments performed vomocytosis faster than the DC 5:1 untreated control (**Fig 5D**). Further, both immune-polarized DCs showed significantly lower time of expulsion than the unpolarized control, with the LPS-treated group having faster expulsion times than the DEX-treated group (**Fig 5E**). In MΦs, the IL4/13-treated condition displayed a longer time to expulsion compared to the control. Additionally, the LPS-treated condition had a significantly faster time of expulsion than the IL4/13-treated group (**Fig 5F)**.

### Different treatments affect number of CN per vomosome

**Fig 6:**
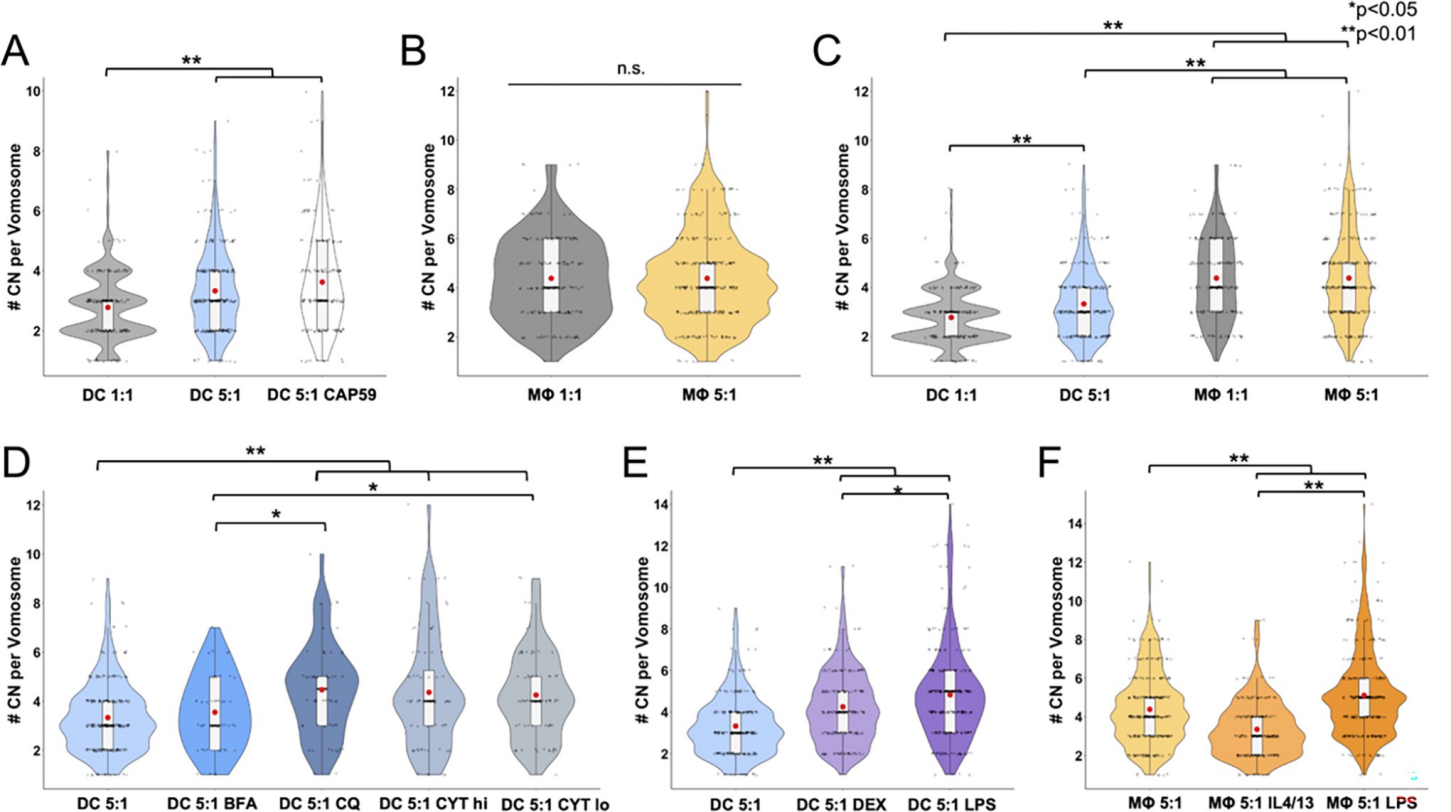
Number of CN per vomosome. Results are displayed in violin box plots with individual dots representing # CN per vomosome for each event. The red circle in each plot represents the mean timing occurrence and the black line in the box plot represents the median. (**A**) Violin plot displaying # CN per DC vomosome compared between 1:1 and 5:1 CN infection ratios, as well as 5:1 infection with a *cap59* CN. (**B**) Violin plot of # CN per MΦ vomosome compared between 1:1 and 5:1 CN infection ratios. (**C**) Violin plot comparing # CN per vomosome between DC and MΦ at different CN infection ratios. (**D**) Violin plot of # CN per vomosome for DCs with a 5:1 CN infection ratio under different drug treated conditions (BFA, CQ, CYT lo, or CYT hi) compared to an untreated control. (**E**) Violin plot of # CN per vomosome for DCs with a 5:1 CN infection as an immature phenotype (untreated), tolerogenic phenotype (DEX treated), and inflammatory phenotype (LPS treated). (**F**) Graph of # CN per vomosome for MΦs with a 5:1 CN infection as an immature M0 phenotype (untreated), tolerogenic M2 phenotype (IL4/13-treated), and inflammatory M1 phenotype (LPS treated) (N≥4, n≥51 for each condition, statistical analyses were performed using an unpaired Mann-Whitney test or a Kruskal-Wallis test corrected for multiple comparisons by FDR using a two-stage linear step-up procedure of Benjamini, Krieger and Yekutieli).

In addition to characterizing the vomocytosis rates and timing of different conditions, the number of CN located in the phagosome before a vomocytosis event, or ‘vomosome’, was also documented and analyzed. Under different infection ratios, the 5:1 condition had a significantly higher average number of CN per vomosome compared to the 1:1 infection ratio for DCs. (**Fig 6A**) However, in MΦs there was no significant difference in CN per vomosome between the 1:1 and 5:1 infection ratios (**Fig 6B**). Notably, both the 1:1 and 5:1 c.p.r. MΦ groups showed higher number of CN located in their vomosomes than both DC 1:1 and DC 5:1 c.p.r. groups (**Fig 6C**). When analyzing drug-treated conditions, the DC 5:1 c.p.r. groups treated with CQ, CYT hi, or CYT lo had a significantly higher number of CN per vomosome than the control. The BFA-treated DC group showed no significant difference in CN per vomosome compared to the untreated control (**Fig 6D**). Finally, immune polarized groups were analyzed for differences in number of CN per vomosome. Both DEX-treated and LPS-treated DCs were observed to have a higher number of CN located in the phagosome prior to a vomocytosis event compared to the unpolarized control. Additionally, the LPS-treated DC group had a higher CN per vomosome count than the DEX-treated group (**Fig 6E**). For polarized MΦs, IL4/13-treated cells displayed a lower CN per vomosome count to the control. Conversely, the LPS-treated group had a higher CN per vomosome average than the control, as well as IL4/13-treated groups. (**Fig 6F**).

## Discussion

Our novel results show that live CN can perform vomocytosis from DCs, to a similar extent exhibited by both MΦs and neutrophils. Here, the occurrence of this phenomenon from DCs was rigorously verified by time-lapse microscopy, confocal imaging, and viability assays. Additionally, the average rate and timing of vomocytosis events from DCs were the same as those from MΦs. On the basis of CD11c, F4/80, and RNA expression, the results confidently support the phenotype and purity of DC cultures and MΦ cultures. Previous literature also corroborates the generation of CD11c+ dendritic cells from non-adherent progenitors treated with GM-CSF [52, 53]. Prior CN-related studies have assumed the bone marrow progenitor differentiation methods used to be considered pure for both DCs and MΦs [9, 18, 21, 40, 47–49]. Using flow cytometry, our results show that DCs have a high CD11c expression. Contrastingly, MΦs showed high expression of the F4/80 integrin. It should be noted that *in vitro*-derived and *in vivo*-isolated DCs and MΦs always show expression of both of these markers, but to differential levels in keeping with our observations. Interestingly, the rates of vomocytosis from these two populations of *in vitro* derived cells are the same, in spite of the differences in F4/80 and CD11c expression. Inferably, *in vitro*-derived DCs are capable vomocytes. Given our results on DC vomocytosis and those published on MΦs and neutrophils, it is likely that some components of this mechanism may be conserved across these cell types derived from common myeloid progenitor cells.

Vomocytosis rates from DCs were observed to have no significant correlation to infection rate or presence of CN capsule. However, DCs infected with *cap59* CN contained a higher (although not significant) number of CN per vomosome compared to DCs infected with the wildtype strain (H99). This is not surprising, as the CN capsule is known to be a potent anti-phagocytic agent that plays a role in reducing uptake by MΦs [54–56] and DCs [57]. Additionally, DCs infected at a 5:1 ratio showed higher amount of CN per vomosome than DCs 1:1 infected, which may be attributed to the larger number of CN that the DCs encountered during the experiment.

When treated with drugs to modify the phagosome (CQ, BFA), DCs displayed substantially lower rates of vomocytosis (although not in the range of significance for the CQ treatment) compared to the untreated control. If treated with an inhibitor of actin polymerization (CYT lo, CYT hi), a similar, but non-significant decrease is shown in vomocytosis rates. Chloroquine is a weak base that passively diffuses into acidic organelles in the cytoplasm, becomes protonated and prevents maturation and fusion of endosomes and lysosomes [58, 59]. Cytochalasin B is an actin polymerization inhibitor that has been demonstrated to induce a release of lysosomal enzymes, modulating lysosomal fusion with phagosomes [60, 61]. Lastly, BFA is involved in the inhibition of the vacuolar ATPase in lysosomes [62]. With respect to CQ, our findings of reduced vomocytosis rates (albeit below significance) conflict with previous studies that have successfully demonstrated this drug to increase vomocytosis from J774 MΦ cell lines [11, 18]. However, *Yang et al* showed that CQ decreases vomocytosis rates from primary murine neutrophils [24]. These discrepancies could be attributed to differences in cell type, as well as source (cell line vs. primary murine cells). Interestingly, the decreased BFA-treated vomocytosis rates we observed from DCs align with observations by *Nicola et al* (from J774 MΦ cells), that this drug treatment reduces vomocytosis rates [18]. On the other hand, neutrophils were shown to have no change in vomocytosis rates after BFA treatment [24]. Again, these differences could be due to cell type, as well as cell source. Overall, our findings indicate that vomocytosis from DCs is indeed inhibited by phagosomal alkalinization—by either the weak base CQ or the ATPase inhibitor BFA. Pertaining to CYT treatment, again there is some disagreement within literature on the effect of this drug on vomocytosis. *Dragotakes et al* observed a decrease in vomocytosis rates of J774 MΦs following treatment with either cytochalasin B or D [46]. Conversely, *Alvarez et al*. saw increased rates of expulsion in the same cell type following cytochalasin D treatment [10]. In neutrophils, cytochalasin D was also seen to increase vomocytosis rates in a study by *Yang et al* [24]. Our observations for DCs show a decrease in vomocytosis rates, albeit insignificant. These trends align with work published by *Dragotakes et al*., but are in conflict with the other two studies. It is possible that cytochalasin B may cause lysosomal fusion in the infected DCs and cripple the CN’s ability to thrive in the phagosome, although our viability data suggests that intracellular CN are still intact during this treatment. The role of CYT in inhibiting actin polymerization may play a more significant role in limiting the phagocyte’s machinery and preventing CN from inducing an expulsion. In investigating average timing of vomocytosis, the CQ-treated and CYT hi-treated vomocytosis events displayed a statistically shorter length of time before expulsion compared to the untreated group. Chloroquine reduces expulsion rates, although slightly below statistical significance, but the CN are released relatively quicker compared to untreated cells. These events may be due to alternate mechanisms such as endosome recycling pathways [63]. Interestingly, within the drug treated groups, the average number of CNs per vomosome were all significantly higher than the untreated group with the exception of the BFA-treated group. It is possible that BFA alkalinization of the phagosome reduces CN dividing ability, as CN has been observed to replicate faster in acidic environments [19, 64].

Treatment of MΦs and DCs with polarization agents did not affect the vomocytosis rates compared to the unpolarized control. This result is contrary to some documented literature for MΦs, as *Gilbert et al*. found that inflammatory-polarized MΦs (via ERK5 inhibition) displayed a higher rate of expulsion than untreated MΦs, shown in both primary human MΦs and J774 cells [9]. Other conflicts exist where *Voelz et al*. showed that anti-inflammatory IL-4 or IL-13 treated MΦs perform a lower rate of vomocytosis compared to an untreated control, also in both primary human MΦs and J774 cells [23]. However, the same study showed that the pro-inflammatory IFN-γ, TNF-α, or IL-17 treated human and J774 MΦs did not have a significantly different vomocytosis rate than unpolarized MΦs, which aligns with our observations. Interestingly, a recent study by *Zhang et al*. found that primary murine MΦs treated with inflammatory extracellular vesicles displayed lower vomocytosis rates [65]—this result contradicts both this study and previous studies. Overall, investigation of the effect of immune phenotype on vomocytosis has been inconclusive. This may be attributed to differences in cell types, polarization procedures, phenotype validation and vomocytosis observation methods. Our results suggest that immune polarization is not a significant influence on vomocytosis rates from MΦs or DCs. However, there was a significant difference in the average time for vomocytosis occurrence for these groups. Cryptococcal cells performed vomocytosis in a significantly shorter time from LPS-treated DCs compared to DEX-treated DCs. Similarly, the average non-lytic exocytosis time of CNs in LPS-treated MΦs was significantly longer in time length compared to that of IL4/13-treated MΦs. This observation is likely due to the differences in molecular and physicochemical characteristics involved in phagolysosomal maturation between activated and tolerogenic phenotypes. For instance, M2 MΦ have been shown to undergo rapid and profound phagosomal acidification relative to M1 MΦs [66]. Moreover, the same study demonstrates that reactive oxygen species (ROS) production is much greater and more sustained in M1 than in M2 phagosomes. Perhaps the mature, ROS and cathepsin-rich phagosome of LPS-treated phagocytes creates an inhospitable environment for CNs, prompting them to escape at a faster rate than in the less harsh anti-inflammatory polarized phagosomes. Additionally, for both DCs and MΦs, the LPS-treated groups displayed higher numbers of CN per vomosome compared to the tolerogenic group. The higher number of CN per vomosome and faster ejection from LPS-treated DCs compared to DEX-treated DCs could suggest a direct correlation between number of CN and ejection time—this same trend was observed from DCs treated with CQ and CYT hi.

## Conclusions

In summary, this study documents the first recorded observation of DCs performing vomocytosis of CN. Moreover, multiple parameters were tested for their effects on vomocytosis including the infection ratio, presence of CN capsule, treatment with drugs, and polarization of phagocyte immune state. Vomocytosis from DCs is independent of infection ratio, CN capsule, or immune polarization phenotype. However, application of the drug BFA that disrupts the phagolysosome significantly inhibits DC vomocytosis. Interestingly, infection ratio, drug treatments, and immune polarization influence the timing of vomocytosis, as well as the number of CN present in the DC vomosome prior to expulsion. Overall, the capability of DCs to expel CNs following ingestion appears similar to that of MΦs based on occurrence rate, timing, and number of CN per vomosome. This finding could help to further elucidate infection dissemination mechanisms in immunocompromised patients, as this phenomenon is clearly conserved between multiple types of phagocytes, including DCs. Finally, more studies are needed to understand the mechanisms involved in vomocytosis from DCs, and moreover determine if there is commonality of processes across all the phagocytic cells that are implicated to perform this behavior. Further, CN is not the only pathogen that has been shown to induce vomocytosis. Therefore, questions on the cohesion of mechanisms across pathogens and phagocytes should be addressed in future investigations.

## Supporting information

S1 Table All significant p-values and statistical methods used for comparisons made in Figs 1–6. (TIF)

S1 Fig Confocal time-lapse microscopy. (TIF)

S2 Fig Viability of CN-infected DCs after 14-hour co-incubation with CNs. (TIF)

S3 Fig Toxicity evaluation of lysosomal maturation and actin polymerization disruptor drugs on CN and DCs. (TIF)

S4 Fig Viability of CN during infection with MΦs or DCs treated with drugs. (TIF)

## References

[1] Rajasingham R. et al., “Global burden of disease of HIV-associated cryptococcal meningitis: an updated analysis,” *The Lancet Infectious Diseases*, vol. 17, no. 8, pp. 873–881, Aug. 2017, doi: 10.1016/S1473-3099(17)30243-8 28483415 PMC5818156

[2] May R. C., Stone N. R. H., Wiesner D. L., Bicanic T., and Nielsen K., “Cryptococcus: from environmental saprophyte to global pathogen,” *Nat Rev Microbiol*, vol. 14, no. 2, Art. no. 2, Feb. 2016, doi: 10.1038/nrmicro.2015.6 26685750 PMC5019959

[3] Cruz-Acuña M., Pacifici N., and Lewis J. S., “Vomocytosis: Too Much Booze, Base, or Calcium?,” *mBio*, Dec. 2019, doi: 10.1128/mBio.02526-19 31874916 PMC6935858

[4] Denham S. T. and Brown J. C. S., “Mechanisms of Pulmonary Escape and Dissemination by Cryptococcus neoformans,” *Journal of Fungi*, vol. 4, no. 1, Art. no. 1, Mar. 2018, doi: 10.3390/jof4010025 29463005 PMC5872328

[5] Sorrell T. C. et al., “Cryptococcal transmigration across a model brain blood-barrier: evidence of the Trojan horse mechanism and differences between Cryptococcus neoformans var. grubii strain H99 and Cryptococcus gattii strain R265,” *Microbes and Infection*, vol. 18, no. 1, pp. 57–67, Jan. 2016, doi: 10.1016/j.micinf.2015.08.017 26369713

[6] Santiago-Tirado F. H., Onken M. D., Cooper J. A., Klein R. S., and Doering T. L., “Trojan Horse Transit Contributes to Blood-Brain Barrier Crossing of a Eukaryotic Pathogen,” *mBio*, Jan. 2017, doi: 10.1128/mBio.02183-16 28143979 PMC5285505

[7] Kechichian T. B., Shea J., and Poeta M. D., “Depletion of Alveolar Macrophages Decreases the Dissemination of a Glucosylceramide-Deficient Mutant of Cryptococcus neoformans in Immunodeficient Mice,” *Infection and Immunity*, Oct. 2007, doi: 10.1128/IAI.00587-07 17664261 PMC2044542

[8] Charlier C., Nielsen K., Daou S., Brigitte M., Chretien F., and Dromer F., “Evidence of a Role for Monocytes in Dissemination and Brain Invasion by Cryptococcus neoformans,” *Infection and Immunity*, Jan. 2009, doi: 10.1128/IAI.01065-08 18936186 PMC2612285

[9] Gilbert A. S. et al., “Vomocytosis of live pathogens from macrophages is regulated by the atypical MAP kinase ERK5,” *Science Advances*, vol. 3, no. 8, p. e1700898, doi: 10.1126/sciadv.1700898 28835924 PMC5559206

[10] Alvarez M. and Casadevall A., “Phagosome Extrusion and Host-Cell Survival after Cryptococcus neoformans Phagocytosis by Macrophages,” *Current Biology*, vol. 16, no. 21, pp. 2161–2165, Nov. 2006, doi: 10.1016/j.cub.2006.09.061 17084702

[11] Ma H., Croudace J. E., Lammas D. A., and May R. C., “Expulsion of Live Pathogenic Yeast by Macrophages,” *Current Biology*, vol. 16, no. 21, pp. 2156–2160, Nov. 2006, doi: 10.1016/j.cub.2006.09.032 17084701

[12] Seoane P. I. and May R. C., “Vomocytosis: What we know so far,” *Cell Microbiol*, vol. 22, no. 2, p. e13145, Feb. 2020, doi: 10.1111/cmi.13145 31730731

[13] Thorn P., Zorec R., Rettig J., and Keating D. J., “Exocytosis in non-neuronal cells,” *Journal of Neurochemistry*, vol. 137, no. 6, pp. 849–859, 2016, doi: 10.1111/jnc.13602 26938142

[14] Tran D. T. and Ten Hagen K. G., “Real-time insights into regulated exocytosis,” *Journal of Cell Science*, vol. 130, no. 8, pp. 1355–1363, Apr. 2017, doi: 10.1242/jcs.193425 28302911 PMC5399783

[15] Wu L.-G., Hamid E., Shin W., and Chiang H.-C., “Exocytosis and Endocytosis: Modes, Functions, and Coupling Mechanisms,” *Annual Review of Physiology*, vol. 76, no. 1, pp. 301–331, 2014, doi: 10.1146/annurev-physiol-021113-170305 24274740 PMC4880020

[16] Johnston S. A. and May R. C., “The Human Fungal Pathogen Cryptococcus neoformans Escapes Macrophages by a Phagosome Emptying Mechanism That Is Inhibited by Arp2/3 Complex-Mediated Actin Polymerisation,” *PLOS Pathogens*, vol. 6, no. 8, p. e1001041, Aug. 2010, doi: 10.1371/journal.ppat.1001041 20714349 PMC2920849

[17] Smith L. M., Dixon E. F., and May R. C., “The fungal pathogen Cryptococcus neoformans manipulates macrophage phagosome maturation,” *Cellular Microbiology*, vol. 17, no. 5, pp. 702–713, 2015, doi: 10.1111/cmi.12394 25394938

[18] Nicola A. M., Robertson E. J., Albuquerque P., da S L. Derengowski, and A. Casadevall, “Nonlytic Exocytosis of Cryptococcus neoformans from Macrophages Occurs In Vivo and Is Influenced by Phagosomal pH,” *mBio*, Aug. 2011, doi: 10.1128/mBio.00167-11 21828219 PMC3150755

[19] Leon-Rodriguez C. M. D., Fu M. S., Çorbali M. O., Cordero R. J. B., and Casadevall A., “The Capsule of Cryptococcus neoformans Modulates Phagosomal pH through Its Acid-Base Properties,” *mSphere*, Oct. 2018, doi: 10.1128/mSphere.00437-18 30355667 PMC6200979

[20] Samantaray S., Correia J. N., Garelnabi M., Voelz K., May R. C., and Hall R. A., “Novel cell-based in vitro screen to identify small-molecule inhibitors against intracellular replication of Cryptococcus neoformans in macrophages,” *International Journal of Antimicrobial Agents*, vol. 48, no. 1, pp. 69–77, Jul. 2016, doi: 10.1016/j.ijantimicag.2016.04.018 27289450 PMC4942879

[21] Fu M. S. et al., “Cryptococcus neoformans urease affects the outcome of intracellular pathogenesis by modulating phagolysosomal pH,” *PLOS Pathogens*, vol. 14, no. 6, p. e1007144, Jun. 2018, doi: 10.1371/journal.ppat.1007144 29906292 PMC6021110

[22] Seoane P. I. et al., “Viral infection triggers interferon-induced expulsion of live Cryptococcus neoformans by macrophages,” *PLoS Pathog*, vol. 16, no. 2, p. e1008240, Feb. 2020, doi: 10.1371/journal.ppat.1008240 32106253 PMC7046190

[23] Voelz K., Lammas D. A., and May R. C., “Cytokine Signaling Regulates the Outcome of Intracellular Macrophage Parasitism by Cryptococcus neoformans,” *Infection and Immunity*, Aug. 2009, doi: 10.1128/IAI.00297-09 19487474 PMC2715691

[24] Yang X., Wang H., Hu F., Chen X., and Zhang M., “Nonlytic exocytosis of Cryptococcus neoformans from neutrophils in the brain vasculature,” *Cell Communication and Signaling*, vol. 17, no. 1, p. 117, Sep. 2019, doi: 10.1186/s12964-019-0429-0 31500648 PMC6734394

[25] Merad M., Sathe P., Helft J., Miller J., and Mortha A., “The Dendritic Cell Lineage: Ontogeny and Function of Dendritic Cells and Their Subsets in the Steady State and the Inflamed Setting,” *Annual Review of Immunology*, vol. 31, no. 1, pp. 563–604, 2013, doi: 10.1146/annurev-immunol-020711-074950 23516985 PMC3853342

[26] Steinman R. M. and Banchereau J., “Taking dendritic cells into medicine,” *Nature*, vol. 449, no. 7161, Art. no. 7161, Sep. 2007, doi: 10.1038/nature06175 17898760

[27] Cabeza-Cabrerizo M., Cardoso A., Minutti C. M., Pereira da Costa M., and Reis e Sousa C., “Dendritic Cells Revisited,” *Annual Review of Immunology*, vol. 39, no. 1, pp. 131–166, 2021, doi: 10.1146/annurev-immunol-061020-053707 33481643

[28] Krishnaswamy J. K., Chu T., and Eisenbarth S. C., “Beyond pattern recognition: NOD-like receptors in dendritic cells,” *Trends in Immunology*, vol. 34, no. 5, pp. 224–233, May 2013, doi: 10.1016/j.it.2012.12.003 23352728 PMC3646908

[29] Hemmi H. and Akira S., “TLR signalling and the function of dendritic cells,” *Chem Immunol Allergy*, vol. 86, pp. 120–135, 2005, doi: 10.1159/000086657 15976491

[30] Förster R. et al., “CCR7 Coordinates the Primary Immune Response by Establishing Functional Microenvironments in Secondary Lymphoid Organs,” *Cell*, vol. 99, no. 1, pp. 23–33, Oct. 1999, doi: 10.1016/s0092-8674(00)80059-8 10520991

[31] Clatworthy M. R., Aronin C. E. P., Mathews R. J., Morgan N. Y., Smith K. G. C., and Germain R. N., “Immune complexes stimulate CCR7-dependent dendritic cell migration to lymph nodes,” *Nat Med*, vol. 20, no. 12, Art. no. 12, Dec. 2014, doi: 10.1038/nm.3709 25384086 PMC4283039

[32] Hampton H. R. and Chtanova T., “Lymphatic Migration of Immune Cells,” *Frontiers in Immunology*, vol. 10, 2019, Accessed: Mar. 03, 2022. [Online]. Available: https://www.frontiersin.org/article/10.3389/fimmu.2019.01168 31191539 10.3389/fimmu.2019.01168 PMC6546724

[33] Tal O. et al., “DC mobilization from the skin requires docking to immobilized CCL21 on lymphatic endothelium and intralymphatic crawling,” *Journal of Experimental Medicine*, vol. 208, no. 10, pp. 2141–2153, Sep. 2011, doi: 10.1084/jem.20102392 21930767 PMC3182054

[34] Russo E. et al., “Intralymphatic CCL21 Promotes Tissue Egress of Dendritic Cells through Afferent Lymphatic Vessels,” *Cell Reports*, vol. 14, no. 7, pp. 1723–1734, Feb. 2016, doi: 10.1016/j.celrep.2016.01.048 26876174

[35] Russo E., Nitschké M., and Halin C., “Dendritic Cell Interactions with Lymphatic Endothelium,” *Lymphatic Research and Biology*, vol. 11, no. 3, pp. 172–182, Sep. 2013, doi: 10.1089/lrb.2013.0008 24044757 PMC3780311

[36] TAKEDA A., SASAKI N., and MIYASAKA M., “The molecular cues regulating immune cell trafficking,” *Proc Jpn Acad Ser B Phys Biol Sci*, vol. 93, no. 4, pp. 183–195, Apr. 2017, doi: 10.2183/pjab.93.012 28413196 PMC5489428

[37] Vono M., Lin A., Norrby-Teglund A., Koup R. A., Liang F., and Loré K., “Neutrophils acquire the capacity for antigen presentation to memory CD4+ T cells in vitro and ex vivo,” *Blood*, vol. 129, no. 14, pp. 1991–2001, Apr. 2017, doi: 10.1182/blood-2016-10-744441 28143882 PMC5383872

[38] BARKER R. N., ERWIG L.-P., HILL K. S. K., DEVINE A., PEARCE W. P., and REES A. J., “Antigen presentation by macrophages is enhanced by the uptake of necrotic, but not apoptotic, cells,” *Clin Exp Immunol*, vol. 127, no. 2, pp. 220–225, Feb. 2002, doi: 10.1046/j.1365-2249.2002.01774.x 11876743 PMC1906351

[39] Randolph G. J., Jakubzick C., and Qu C., “Antigen presentation by monocytes and monocyte-derived cells,” *Curr Opin Immunol*, vol. 20, no. 1, pp. 52–60, Feb. 2008, doi: 10.1016/j.coi.2007.10.010 18160272 PMC2408874

[40] Kelly R. M., Chen J., Yauch L. E., and Levitz S. M., “Opsonic Requirements for Dendritic Cell-Mediated Responses to Cryptococcus neoformans,” *Infection and Immunity*, vol. 73, no. 1, pp. 592–598, Jan. 2005, doi: 10.1128/IAI.73.1.592-598.2005 15618199 PMC539000

[41] Hole C. R., Bui H., Wormley F. L., and Wozniak K. L., “Mechanisms of dendritic cell lysosomal killing of Cryptococcus,” *Scientific reports*, vol. 2, no. 1, pp. 1–9, 2012. doi: 10.1038/srep00739 23074646 PMC3472389

[42] Artavanis-Tsakonas K., Love J. C., Ploegh H. L., and Vyas J. M., “Recruitment of CD63 to Cryptococcus neoformans phagosomes requires acidification,” *Proceedings of the National Academy of Sciences*, vol. 103, no. 43, pp. 15945–15950, 2006. doi: 10.1073/pnas.0607528103 17043215 PMC1635107

[43] Allen R. P., Bolandparvaz A., Ma J. A., Manickam V. A., and Lewis J. S., “Latent, Immunosuppressive Nature of Poly(lactic-co-glycolic acid) Microparticles,” *ACS Biomater*. *Sci*. *Eng*., vol. 4, no. 3, pp. 900–918, Mar. 2018, doi: 10.1021/acsbiomaterials.7b00831 30555893 PMC6290919

[44] Zhang X., Goncalves R., and Mosser D. M., “The Isolation and Characterization of Murine Macrophages,” *Current Protocols in Immunology*, vol. 83, no. 1, p. 14.1.1–14.1.14, 2008, doi: 10.1002/0471142735.im1401s83 19016445 PMC2834554

[45] Lutz M. B. et al., “An advanced culture method for generating large quantities of highly pure dendritic cells from mouse bone marrow,” *Journal of Immunological Methods*, vol. 223, no. 1, pp. 77–92, Feb. 1999, doi: 10.1016/s0022-1759(98)00204-x 10037236

[46] Dragotakes Q., Fu M. S., and Casadevall A., “Dragotcytosis: Elucidation of the Mechanism for Cryptococcus neoformans Macrophage-to-Macrophage Transfer,” *The Journal of Immunology*, Mar. 2019, doi: 10.4049/jimmunol.1801118 30877168 PMC6495551

[47] Tanaka M. et al., “Toll-Like Receptor 9-Dependent Activation of Bone Marrow-Derived Dendritic Cells by URA5 DNA from Cryptococcus neoformans,” *Infect Immun*, vol. 80, no. 2, pp. 778–786, Feb. 2012, doi: 10.1128/IAI.05570-11 22104112 PMC3264295

[48] Nakamura K. et al., “Deoxynucleic Acids from Cryptococcus neoformans Activate Myeloid Dendritic Cells via a TLR9-Dependent Pathway,” *The Journal of Immunology*, vol. 180, no. 6, pp. 4067–4074, Mar. 2008, doi: 10.4049/jimmunol.180.6.4067 18322216

[49] Grahnert A. et al., “IL-4 Receptor-Alpha-Dependent Control of Cryptococcus neoformans in the Early Phase of Pulmonary Infection,” *PLoS One*, vol. 9, no. 1, p. e87341, Jan. 2014, doi: 10.1371/journal.pone.0087341 24475277 PMC3903725

[50] dos Anjos Cassado A., “F4/80 as a major macrophage marker: the case of the peritoneum and spleen,” *Macrophages*, pp. 161–179, 2017. 10.1007/978-3-319-54090-0_7 28455709

[51] Pépin E., Goutet M., and Ban M., “Murine bone marrow-derived dendritic cells as a potential in vitro model for predictive identification of chemical sensitizers,” *Toxicology letters*, vol. 175, no. 1–3, pp. 89–101, 2007. doi: 10.1016/j.toxlet.2007.09.012 18006254

[52] Wan H. and Dupasquier M., “Dendritic cells in vivo and in vitro,” *Cellular & Molecular Immunology*, Jan. 2005, Accessed: May 25, 2022. [Online]. Available: https://repub.eur.nl/pub/10396/ 16212908

[53] Rogers P. B., Driessnack M. G., and Schwartz E. H., “Analysis of the developmental stages, kinetics, and phenotypes exhibited by myeloid cells driven by GM-CSF in vitro,” *PLOS ONE*, vol. 12, no. 7, p. e0181985, Jul. 2017, doi: 10.1371/journal.pone.0181985 28750033 PMC5531556

[54] Bolaños B. and Mitchell T. G., “Phagocytosis of Cryptococcus neoformans by rat alveolar macrophages,” *Journal of Medical and Veterinary Mycology*, vol. 27, no. 4, pp. 203–217, Jul. 1989, doi: 10.1080/02681218980000291 2677298

[55] Levitz S. M. and DiBenedetto D. J., “Paradoxical role of capsule in murine bronchoalveolar macrophage-mediated killing of Cryptococcus neoformans,” *J Immunol*, vol. 142, no. 2, pp. 659–665, Jan. 1989. 2521352

[56] Granger D. L., Perfect J. R., and Durack D. T., “Virulence of Cryptococcus neoformans. Regulation of capsule synthesis by carbon dioxide.,” *J Clin Invest*, vol. 76, no. 2, pp. 508–516, Aug. 1985, doi: 10.1172/JCI112000 3928681 PMC423853

[57] Vecchiarelli A., Pietrella D., Lupo P., Bistoni F., McFadden D. C., and Casadevall A., “The polysaccharide capsule of Cryptococcus neoformans interferes with human dendritic cell maturation and activation,” *Journal of Leukocyte Biology*, vol. 74, no. 3, pp. 370–378, 2003, doi: 10.1189/jlb.1002476 12949240

[58] Halcrow P. W., Geiger J. D., and Chen X., “Overcoming chemoresistance: Altering pH of cellular compartments by chloroquine and hydroxychloroquine,” *Frontiers in Cell and Developmental Biology*, vol. 9, p. 170, 2021. doi: 10.3389/fcell.2021.627639 33634129 PMC7900406

[59] Xia M.-C., Cai L., Zhang S., and Zhang X., “A cell-penetrating ratiometric probe for simultaneous measurement of lysosomal and cytosolic pH change,” *Talanta*, vol. 178, pp. 355–361, 2018. doi: 10.1016/j.talanta.2017.09.044 29136833

[60] Zurier R. B., Hoffstein S., and Weissmann G., “Cytochalasin B: effect on lysosomal enzyme release from human leukocytes,” *Proceedings of the National Academy of Sciences*, vol. 70, no. 3, pp. 844–848, 1973. 10.1073/pnas.70.3.844 PMC433372 4351807

[61] Koza E. P., Wright T. E., and Becker E. L., “Lysosomal enzyme secretion and volume contraction induced in neutrophils by cytochalasin B, chemotactic factor and A23187,” *Proceedings of the Society for Experimental Biology and Medicine*, vol. 149, no. 2, pp. 476–479, 1975. doi: 10.3181/00379727-149-38831 1098054

[62] Tapper H. and Sundler R., “Bafilomycin A1 inhibits lysosomal, phagosomal, and plasma membrane H+‐ATPase and induces lysosomal enzyme secretion in macrophages,” *Journal of cellular physiology*, vol. 163, no. 1, pp. 137–144, 1995. doi: 10.1002/jcp.1041630116 7896890

[63] Hsu V. W. and Prekeris R., “Transport at the recycling endosome,” *Current Opinion in Cell Biology*, vol. 22, no. 4, pp. 528–534, Aug. 2010, doi: 10.1016/j.ceb.2010.05.008 20541925 PMC2910225

[64] DeLeon-Rodriguez C. M. and Casadevall A., “Cryptococcus neoformans: Tripping on Acid in the Phagolysosome,” *Frontiers in Microbiology*, vol. 7, 2016, Accessed: Mar. 08, 2022. [Online]. Available: https://www.frontiersin.org/article/10.3389/fmicb.2016.00164 26925039 10.3389/fmicb.2016.00164 PMC4756110

[65] Zhang L. et al., “Cryptococcus neoformans-Infected Macrophages Release Proinflammatory Extracellular Vesicles: Insight into Their Components by Multi-omics,” *mBio*, vol. 12, no. 2, pp. e00279–21, doi: 10.1128/mBio.00279-21 33785616 PMC8092229

[66] Canton J., Khezri R., Glogauer M., and Grinstein S., “Contrasting phagosome pH regulation and maturation in human M1 and M2 macrophages,” *Mol Biol Cell*, vol. 25, no. 21, pp. 3330–3341, Nov. 2014, doi: 10.1091/mbc.E14-05-0967 25165138 PMC4214780

