# Supplementary figures and images for: Vomocytosis of Cryptococcus neoformans cells from murine, bone marrow-derived dendritic cells
Source: PLoS One. 2023 Mar 16;18(3):e0280692. doi: 10.1371/journal.pone.0280692 (PMC10019626; doi:10.1371/journal.pone.0280692)

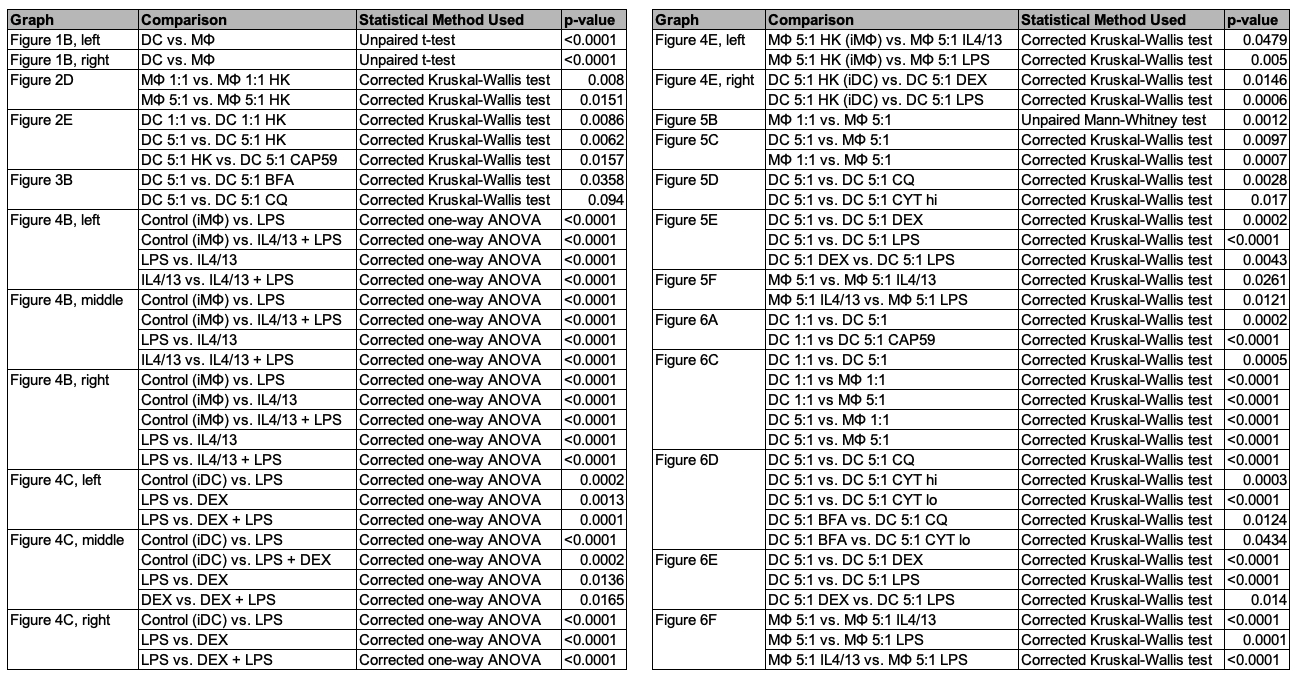

Supplement: S1 Table — (TIF) [file pone.0280692.s001.tif]

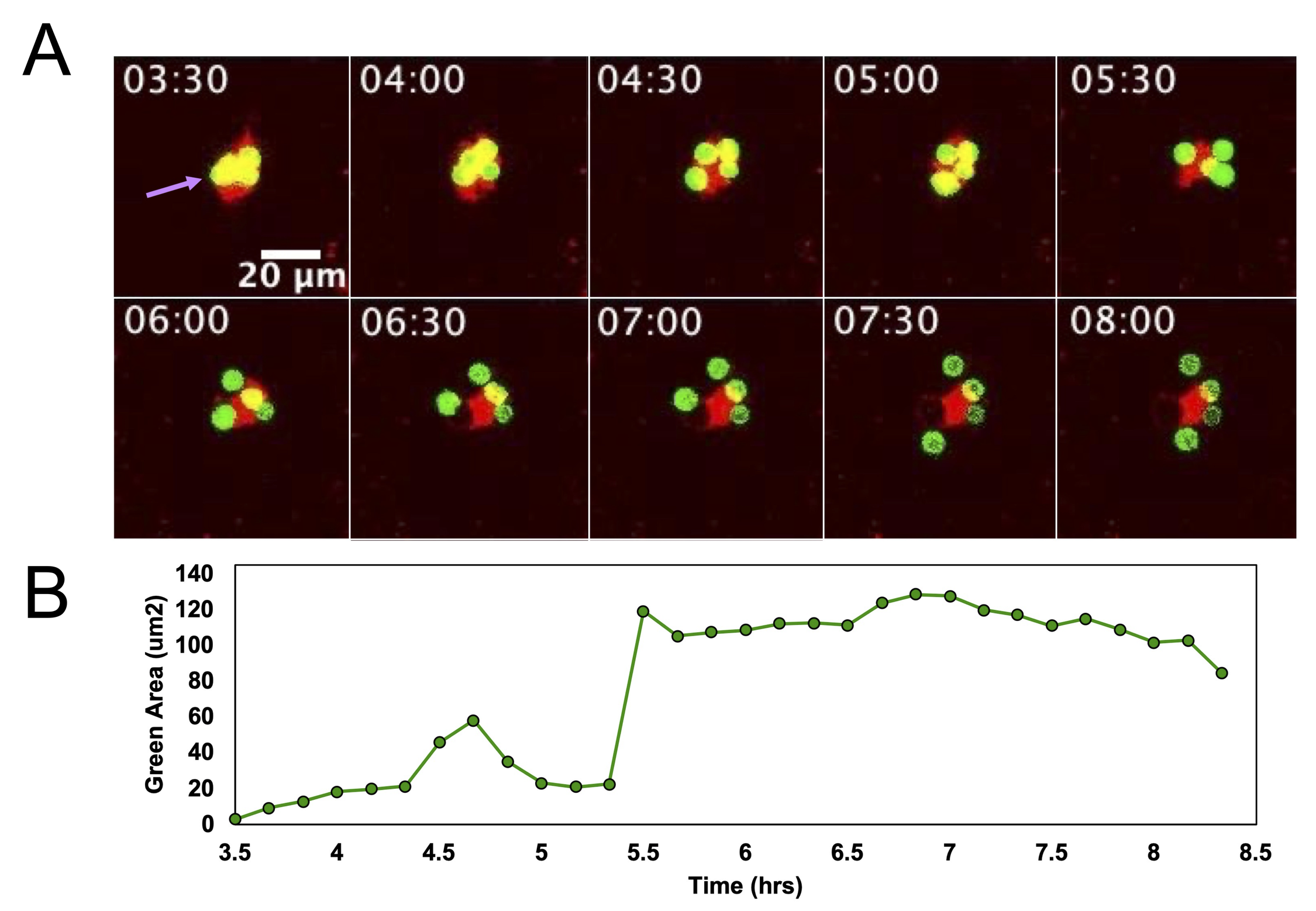

Supplement: S1 Fig — (TIF) [file pone.0280692.s002.tif]

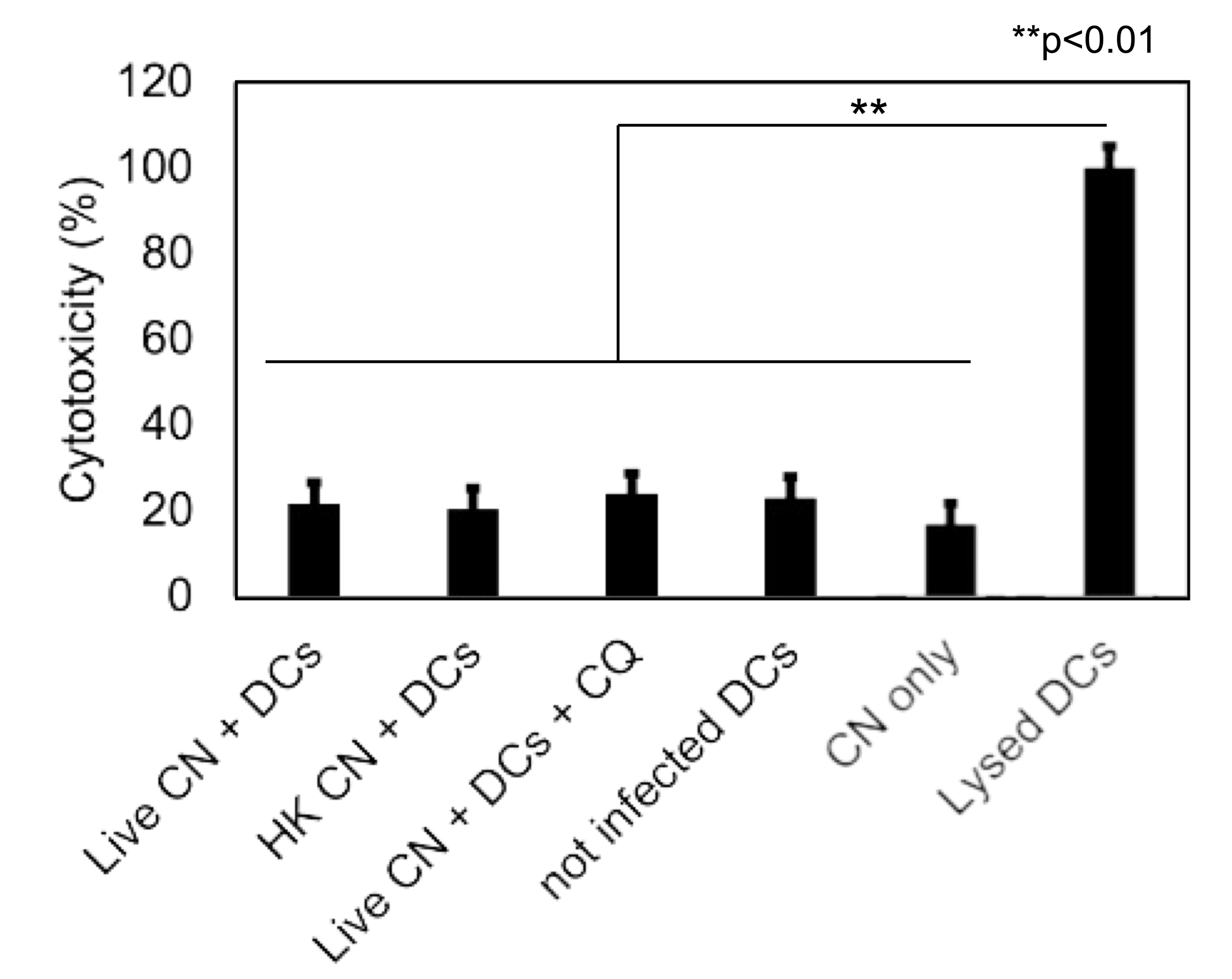

Supplement: S2 Fig — (TIF) [file pone.0280692.s003.tif]

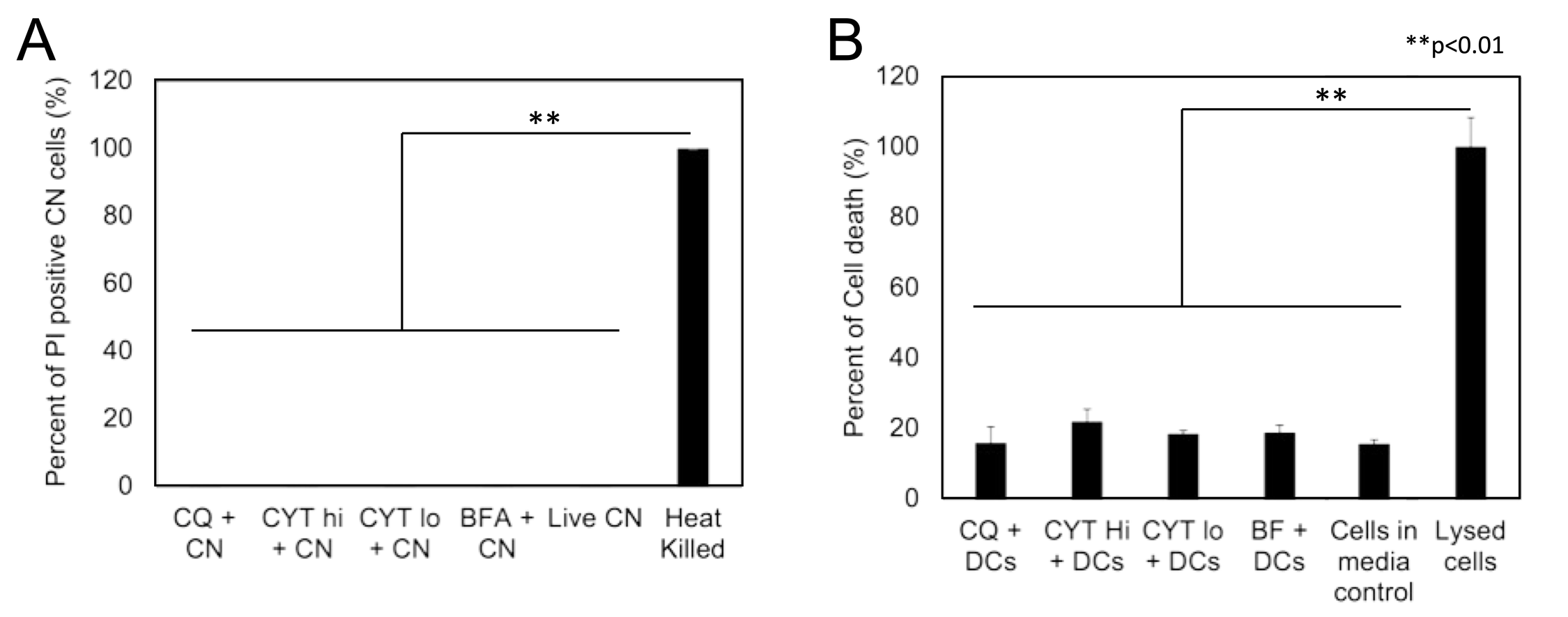

Supplement: S3 Fig — (TIF) [file pone.0280692.s004.tif]

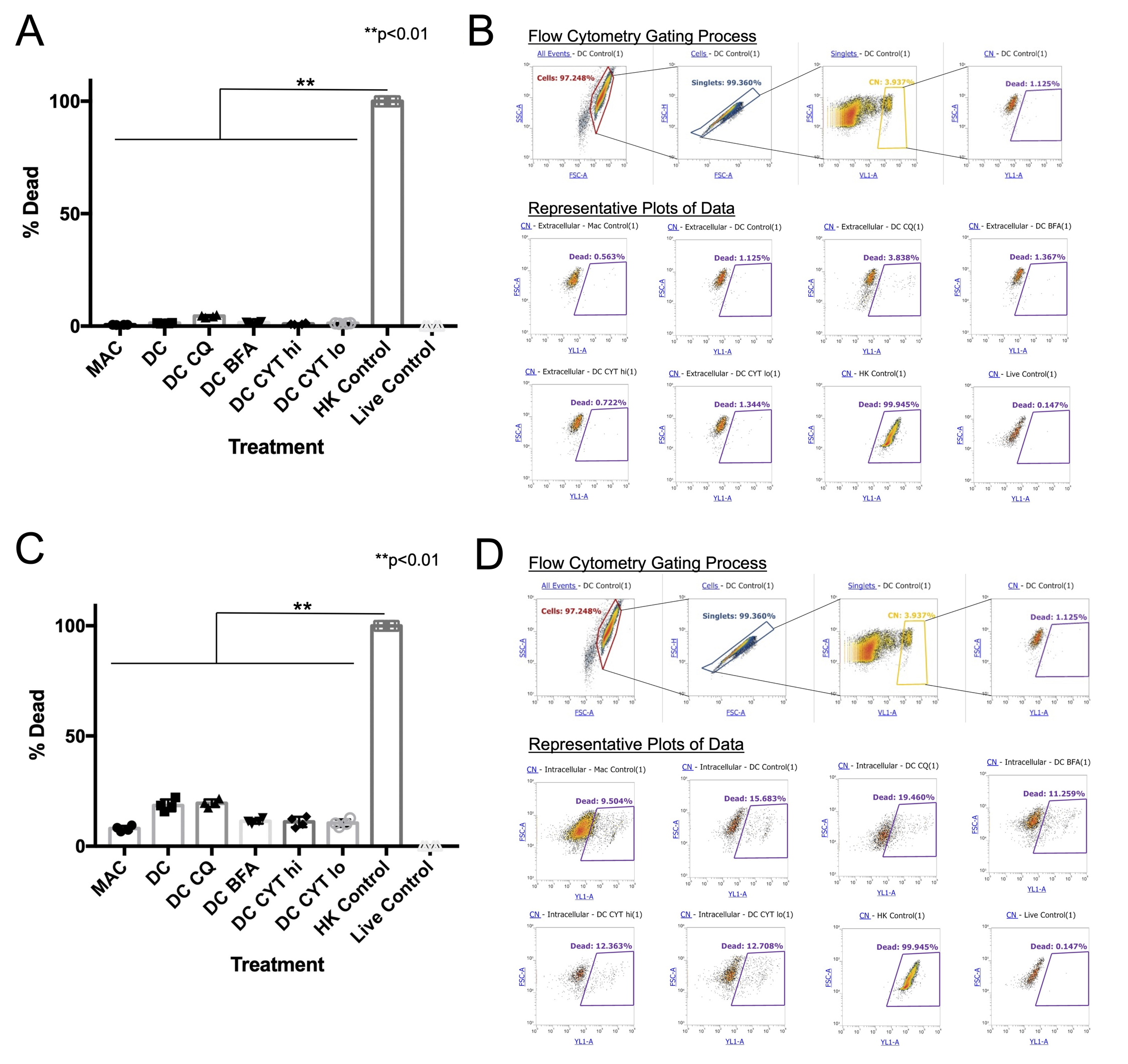

Supplement: S4 Fig — (TIF) [file pone.0280692.s005.tif]
